# Simulation of Propofol Target-Controlled Infusion up to Time of Delivery in Cesarean Section: A Bench Study

**DOI:** 10.3390/jcm14207234

**Published:** 2025-10-14

**Authors:** Ilja Osthoff, Monica Soare, Giulio Barana, Wieland Sell, JoEllen Welter, Alexander Dullenkopf

**Affiliations:** 1 Institute for Anesthesia, Spital Thurgau, 8500 Frauenfeld, Switzerland; ilja.osthoff@stgag.ch (I.O.); monica.soare@balgrist.ch (M.S.); giulio.barana@stgag.ch (G.B.); joellen.welter@stgag.ch (J.W.); 2 Department of Obstetrics and Gynecology, Spital Thurgau Frauenfeld, 8500 Frauenfeld, Switzerland; wieland.sell@stgag.ch

**Keywords:** cesarean delivery, general anesthesia, propofol, target-controlled infusion

## Abstract

**Background/Objectives**: General anesthesia is occasionally required for cesarean delivery (CD). Propofol target-controlled infusion (TCI) enables dosing based on pharmacokinetic modeling. During the transition from induction to maintenance, infusion pauses. This simulation study assessed propofol from induction to delivery and the proportion of deliveries estimated during this pause. **Methods**: Surgical data from women undergoing CD were compiled, and the demographics were entered into a TCI pump using the Schnider model. Effect-site targets (6 and 8 mcg/mL) were simulated for induction, followed by 2.5 mcg/mL for maintenance. Outcomes were estimated propofol dose from induction to delivery and timing of delivery relative to infusion pause. **Results**: Among 50 women, the estimated mean propofol dose from induction to delivery was 19 ± 22 mg (0.2 ± 0.3 mg/kg) at 6 mcg/mL and 13 ± 17 mg (0.2 ± 0.2 mg/kg) at 8 mcg/mL. Delivery occurred during the infusion pause in 40% and 50% of cases, and it was more often in emergency than elective procedures. Emergency status, but not age or body mass index, predicted delivery during the pause. **Conclusions**: Standardized TCI with reduced effect-site targets for maintenance resulted in modest propofol administration between induction and delivery. These findings require confirmation in clinical studies, where dosing should be guided by depth-of-anesthesia monitoring.

## 1. Introduction

Neuraxial anesthesia, including spinal, epidural, and combined spinal–epidural techniques, is now the preferred method of care for cesarean delivery (CD) due to its safety, efficacy, and maternal and neonatal benefits [1,2,3]. From the maternal perspective, regional anesthesia enables conscious participation in childbirth while avoiding the risks associated with general anesthesia, including airway complications, pulmonary aspiration, and postoperative nausea and vomiting (PONV). The neonatal advantages include minimal drug transfer to the baby, potentially faster neonatal adaptation, and decreased risks of respiratory depression, hypotonia, and the need for resuscitation [4]. Nevertheless, general anesthesia may be necessary in emergencies, when neuraxial blocks are contraindicated, if the patient refuses neuraxial anesthesia or if neuraxial anesthesia is insufficient. In such cases, careful anesthetic management is critical. General anesthesia for CD must balance several competing priorities: achieving adequate maternal depth of anesthesia, maintaining utero-placental perfusion, minimizing neonatal exposure, and allowing for rapid emergence post-delivery.

Anesthesia for cesarean delivery may be induced with propofol, a widely used short-acting intravenous anesthetic agent, or sodium thiopental [5,6,7,8,9]. Ketamine may be used in certain circumstances [10]. When propofol is used for induction, there is concern that it may cause maternal hypotension, potentially compromising placental perfusion [10]. Additionally, propofol crosses the placenta, raising concerns about neonatal depression [11]. Nonetheless, its widespread familiarity and favorable pharmacokinetic profile support its use when administered in controlled doses [5]. Anesthesia is traditionally maintained with an inhalational agent, such as sevoflurane [8,12]. Following delivery, some anesthetists switch from sevoflurane to propofol for maintenance, aiming to reduce the risk of uterine relaxation resulting in excessive bleeding [13,14].

Target-controlled infusion (TCI) is an advanced method for administering intravenous agents, such as propofol, by setting a target for plasma (Cpt) or effect-site (brain, Cet) concentration [15]. A computer-aided infusion pump individualizes dosing based on a pharmacokinetic/pharmakodynamic (PK/PD) model; for example, the Schnider model accounts for a patient’s sex, age, height, and weight [16]. To quickly achieve the selected Cet, the TCI pump selects an initial dose for plasma concentration (Cp) that exceeds the Cet. For instance, if Cet is set to 8 mcg/mL, the initial Cp will rise to approximately 20 mcg/mL before the infusion rate is reduced. A steady state is eventually achieved as the propofol redistributes from the plasma to the effect site. Conversely, a reduction in Cet is typically accomplished by temporarily pausing the infusion. Depending on the PK/PD model selected and individual patient characteristics, a pause in propofol infusion may persist for several minutes. Given that cesarean delivery can occur within this timeframe, it is plausible that, following the induction bolus, little to no supplemental propofol would be administered prior to delivery.

Propofol TCI is widely used in non-obstetric anesthesia, and anesthetists are familiar with its principles and advantages. However, actual TCI dosing must be guided by depth-of-anesthesia monitoring, such as raw or processed EEG [17].

This bench study was designed to simulate propofol TCI in women undergoing cesarean delivery. The primary objectives were (i) to estimate the propofol dose that would be administered up to the time of delivery and (ii) to determine the proportion of deliveries that would occur during the infusion pause that follows reduction of the effect-site target concentration after induction. We hypothesized that, given the typical duration of the procedure, the additional amount of propofol beyond induction would be low.

## 2. Materials and Methods

This bench study was conducted in accordance with the guidelines detailed in the Declaration of Helsinki. The local ethics committee (Ethics Committee East Switzerland; EKOS 24/026; 22 February 2024) granted an exemption from research ethics review for this modeling-based analysis, which was designed to inform clinical practice improvements. This study was conducted at an acute care hospital in northeast Switzerland with approximately 250 beds. The hospital serves a largely rural community of about 150,000 inhabitants and manages approximately 1,200 inpatient childbirths annually.

Data from women undergoing an elective or emergency cesarean delivery at the institution during 2024 were screened for inclusion. To assess eligibility, we identified patients who had provided written informed consent for the use of their data in research (the institution’s general consent form is approved by the local human research ethics committee for a broad range of studies). Only data from patients who consented to the use of their information were included in the analysis. We excluded patients with multiple pregnancies. All indications for cesarean delivery were considered eligible. Furthermore, the surgery could have been performed under neuraxial anesthesia or general anesthesia. This study used a convenience sample from the eligible population, and no a priori power analysis was performed to determine the required sample size.

The patient’s age, height, and weight at the time of the cesarean delivery were retrieved from the hospital’s electronic medical records system (KISIM; Cistec, Zurich, Switzerland). Additionally, we determined whether the surgery was elective (planned in advance) or an emergency (unplanned) procedure. Lastly, we determined the time from incision to delivery.

The information gathered from the medical records was then programmed into the propofol TCI system (Alaris Infusion Pump; BD, Alschwil, Switzerland). We used the Schnider model, a PK/PD framework for propofol administration designed to predict drug distribution, metabolism, and elimination, enabling precise dosing to achieve a targeted plasma or effect-site concentration [18]. In this study, we investigated three distinct effect-site concentration targets: 4, 6, and 8 mcg/mL.

For the simulation, we initiated the induction, and the resulting virtual plasma level was monitored. When the plasma level reached its peak, the cumulative propofol dose administered up to the point was recorded. It was assumed that, in a real clinical scenario, one minute after reaching the plasma peak (t0), the patient would be adequately anesthetized for tracheal intubation and surgical incision. At this stage, the effect-site target concentration was adjusted to a maintenance level of 2.5 mcg/mL, in accordance with our institution’s standard protocol for non-obstetric surgery.

The time of delivery (tD) was obtained from the anesthesia report in the medical records. The interval from plasma peak (t0) to reactivation of the infusion pump—required to stabilize the maintenance effect-site target concentration—was recorded. Additionally, the total propofol dose administered was documented at one-minute intervals for ten minutes. Using the recorded duration from incision to delivery (t0 to tD), the total cumulative propofol dose that would have been administered to the patient was calculated, and it was determined whether delivery would have occurred while the infusion pump was in stop mode.

Descriptive statistics were used to summarize baseline characteristics and simulation outcomes. Continuous variables are presented as means with standard deviations (SD), and categorical variables are reported as frequencies and percentages with 95% confidence intervals (CI). Comparisons between elective and emergency cesarean deliveries were performed using two-sample *t*-tests. Differences in proportions were assessed using chi-square or Fisher’s exact tests, as appropriate.

To address potential confounding, multivariable logistic regression analyses were performed for each induction effect-site target (4, 6, and 8 mcg/mL) to evaluate whether delivery occurred during the infusion pause (binary outcome). Predictor variables were surgical urgency (emergency vs. elective), maternal age, and body mass index (BMI). BMI was used rather than height and weight to avoid collinearity. Results are reported as odds ratios (OR) with 95% confidence intervals. Differences in incision-to-delivery time were examined using multivariable linear regression with the same predictors. Model fit for linear regression was assessed using R^2^. Statistical significance was set at *p* < 0.05. All analyses were performed in Stata version 15 (StataCorp, College Station, TX, USA).

## 3. Results

Of the 63 pregnant women assessed for inclusion, 50 patients were eligible and included in the analysis. The reasons for exclusion were the following: 3 were missing a consent form, and 10 denied use of their data for research purposes. At the time of the cesarean delivery, the mean age of the women was 32.3 ± 4.3 years. The mean height and weight were 164.8 ± 5.9 cm and 82.8 ± 17.5 kg, respectively. Consequently, the mean BMI was 30.5 ± 6.6 kg/m^2^. Twenty-nine cases (58%) were elective procedures.

The mean time from incision to delivery for the entire cohort was 6.1 ± 3.2 min. The duration was longer for elective procedures (7.2 ± 3.3 min) than the emergency cases (4.7 ± 2.6 min). The mean difference was 2.5 min (95% CI 0.7–4.2; *p* = 0.007). Overall, the mean propofol dose at t0 (one minute after peak of plasma concentration) was 107.0 ± 26.6 mg (1.3 ± 0.1 mg/kg) when the effect-site target was set to 4 mcg/mL. When Cet was set to 6 mcg/mL, the mean dose increased to 161.3 ± 40.9 mg (1.9 ± 0.1 mg/kg). Similarly, when the Cet was established at 8 mcg/mL, the mean dose further increased to 216.8 ± 55.9 mg (2.6 ± 0.1 mg/kg). No significant differences were observed between the elective and emergency cases at any of these three effect-site targets (*p* = 0.5798, *p* = 0.5923, and *p* = 0.5814, respectively).

The amounts of propofol administered after induction to delivery, along with data stratified by elective and emergency cases, are presented in Table 1. For the entire cohort, the mean propofol dose at delivery was 139.8 ± 42.0 mg (1.7 ± 0.3 mg/kg) when the effect-site target was set at 4 mcg/mL. The mean difference between the elective and emergency cases was 29.2 mg (95% CI 5.9–52.4; *p* = 0.015). The mean dose increased to 180.1 ± 46.3 mg (2.2 ± 0.3 mg/kg) at 6 mcg/mL, with a mean difference of 23.5 mg (95% CI −2.8–49.9; *p* = 0.079). For simulations with an induction effect-site target of 8 mcg/mL, the mean propofol dose was 230.2 ± 58.3 mg (2.8 ± 0.2 mg/kg), and the mean difference between the two types of CDs was 20.7 mg (95% CI−13.0–54.4; *p* = 0.223).

After reducing the effect-site target at t_0_ to the anesthesia maintenance level of 2.5 mcg/mL, the infusion pump stopped administering propofol for 175.3 ± 12.7 s after induction with a Cet of 4 mcg/mL. The pause duration increased to 279.7 ± 33.8 s at 6 mcg/mL and 355.3 ± 56.1 s at 8 mcg/mL. The mean propofol doses administered at each time point are presented in Table 2. Comparisons between the elective and emergency procedures showed no significant differences in pause duration at any of the three induction targets (*p* = 0.7939, *p* = 0.7084, and *p* = 0.8408, respectively).

With an induction effect-site target of 4 mcg/mL, delivery would have occurred during the period from t0 to resumption of propofol administration in 16% of cases (95% CI 8–29%), corresponding to 8 out of 50 women. The proportions were similar between emergency (19%) and elective (14%) procedures (*p* = 0.6169). At 6 mcg/mL, delivery would have taken place during the pause in 40% of cases (95% CI 27–54%; 20/50), with a higher rate in emergency (62%) compared to elective (24%) procedures (*p* = 0.010). At 8 mcg/mL, the overall proportion increased to 50% of cases (95% CI 36–64%; 25/50), which was also higher in emergency (71%) than elective (34%) procedures (*p* = 0.021).

Multivariable logistic regression analyses were performed to evaluate whether emergency status, maternal age, or BMI predicted the likelihood of delivery occurring during the infusion pause. At an induction target of 4 mcg/mL, none of the predictors reached significance. At 6 mcg/mL, emergency deliveries were nearly six times more likely to occur during the pause compared to elective procedures (OR 5.80, 95% CI 1.57–21.40; *p* = 0.008), whereas age (*p* = 0.078) and BMI (*p* = 0.995) were not significant. Similarly, at 8 mcg/mL, emergency procedures remained a predictor of delivery in the pause (OR 5.58, 95% CI 1.51–20.65; *p* = 0.010), while age (*p* = 0.129) and BMI (*p* = 0.245) showed no significant associations.

In multivariable linear regression, emergency cesarean delivery was associated with an incision-to-delivery interval that was, on average, 2.4 min shorter (95% CI −4.1–−0.7; *p* = 0.007) after adjusting for maternal age and BMI. Neither maternal age (*p* = 0.097) nor BMI (*p* = 0.445) were significant predictors, and the model explained approximately 20% of the variability in incision-to-delivery intervals (R^2^ = 0.20).

## 4. Discussion

For this bench study, where target-controlled infusion (TCI) of propofol was simulated using data from 50 women undergoing an elective or emergency cesarean delivery, we examined the amount of propofol administered from induction to delivery at three distinct induction effect-site targets (all of which were reduced to a maintenance concentration of 2.5 mcg/mL after induction): 4, 6, and 8 mcg/mL. The primary finding was that, following induction and until simulated delivery, only moderate doses of propofol—defined as less than 0.5 mg/kg—would have been administered, if any at all. Deliveries were estimated to occur during the infusion pause in 16%, 40%, and 50% of cases at 4, 6, and 8 mcg/mL, respectively, with higher proportions in emergency compared to elective procedures at the higher induction targets.

In cases where general anesthesia is necessary for cesarean deliveries, there are no universally established guidelines for its administration [19]. Traditionally, general anesthesia for cesarean deliveries involves intravenous induction, followed by maintenance with inhalational anesthetics [5,10,20]. This approach contrasts with modern non-obstetric anesthesia, which is mostly induced and maintained by propofol. Propofol is considered more sustainable and environmentally friendly than inhalational agents, and it is associated with reduced uterine bleeding [5,14,21]. In the target-controlled infusion of propofol, the administration mimics a bolus dose for induction, followed by an adapted continuous infusion to maintain the target plasma or effect-site concentration [22]. The infusion rate is gradually reduced to compensate for propofol accumulation. When transitioning from induction to maintenance, that is, when the target concentration is lowered, the infusion may be temporarily stopped.

At our institution, in non-obstetric surgery, propofol TCI is routinely initiated with an induction effect-site target of 6 to 8 mcg/mL. The Schnider TCI model achieves this by administering a propofol dose that produces a plasma peak concentration that is approximately three times the effect-site target. Based on clinical experience, the bispectral index EEG value (BIS) usually falls below 60—indicative of a low probability of recall—approximately one minute after the plasma peak is reached. After induction, the Cet is reduced to 2.5 mcg/mL. We adopted these settings in this bench study under the assumption that, in a real clinical scenario, tracheal intubation would be performed, followed by surgical incision at the corresponding time point. Compared to a traditional bolus induction of 1.5 to 2.8 mg/kg propofol [3,5], the induction effect-site target of 6 mcg/mL in our study corresponded to a bolus of 1.9 mg/kg (increasing to 2.6 mg/kg for effect-site target of 8 mcg/mL). Conversely, selecting a lower effect-site target of 4 mcg/mL would have resulted in a bolus of 1.3 mg/kg, which, without co-medication, may not be sufficient to prevent intraoperative awareness in a stressed patient.

The total propofol dose administered up to delivery after an induction effect-site target of 6 mcg/mL would have been only 19 mg higher than the baseline dose (and 9 mg higher in emergency cases). This incremental dose should be considered in relation to the amount of inhalational agent typically required to maintain anesthesia in a conventional anesthetic technique. Importantly, both propofol and volatile anesthetics, such as sevoflurane, cross the placental barrier and may potentially affect the newborn. Interestingly, after induction with a Cet of 6 mcg/mL, no additional propofol would have been administered after induction until delivery in 40% of cases (and 62% of emergency cases).

Our bench study has limitations that warrant discussion. First, our simulation focused on propofol to isolate its impact. A more comprehensive simulation incorporating processed EEG monitoring and multi-drug pharmacology would provide a more realistic representation. Performing calculations of different timings of incision to delivery for fictional patients with different body characteristics would have been a possibility. However, our goal was to show a representation of a range of clinical scenarios. By utilizing real patient data, our study captured natural variability in surgical times but involved elective and emergency cases that could have implications for real-patient anesthetics.

While our project may rely on a rather simplistic approach, during individual anesthetics, it would be necessary to monitor and adjust effect-site targets dynamically and administer additional medications as needed, based on the patient’s response. Additionally, we assumed that tracheal intubation and surgical incision occurred at a fixed time point. However, in clinical practice, this interval can vary in duration, potentially affecting parameters such as anesthetic depth at the time of delivery. This variability is clinically relevant, as preventing intraoperative awareness under anesthesia is critical yet challenging in patients undergoing cesarean deliveries [5,19,23]. Continuous depth of anesthesia monitoring and, if necessary, supplementation of anesthesia after delivery, should be standard practice [17]. Moreover, pregnancy is associated with increased volume of distribution, meaning that a pregnant woman cannot be equated to a non-pregnant woman of the same age, weight, and height. Another limitation of this bench study is that the Schnider model has not been validated in late pregnancy. An additional drawback to our study is that it was restricted to the Schnider TCI model as implemented in the Alaris infusion pump. Future research should compare induction dosing and dose reduction behavior between the Schnider and other models (e.g., Marsh, Eleveld).

Although our regression model identified surgical urgency as a predictor of delivery during the infusion pause, its explanatory power was modest (R^2^ = 0.20 for incision-to-delivery time). This suggests that unmeasured factors—such as surgical complexity, fetal urgency, operator experience, and operating room logistics—may have also contributed to variability. Our study was further limited by the relatively small, single-center sample size, and residual confounding cannot be excluded, as comorbidities and anesthesia techniques were not included. Most importantly, these results represent modeled estimates rather than observed clinical drug administration and should be interpreted with caution until validated in prospective patient studies.

In conclusion, using target-controlled infusion technique for administering propofol-based anesthetics for cesarean delivery with a standardized induction protocol followed by a reduced effect-site target for maintenance would likely result in only moderate additional propofol administration from induction to delivery. Depth of anesthesia monitoring is mandatory when performing propofol-based anesthesia in such cases.

## Figures and Tables

**Table 1:** Propofol dose by surgical group (based on effect-site concentration levels at induction and adjustment to maintenance (2.5 mcg/mL) at t0): (i) at delivery (t_D_) and (ii) from induction to delivery (t_0_ to t_D_).

Propofol Dose *:	t_D_			t_0_ to t_D_		
	Total Cohort	Elective Surgery	Emergency Surgery	Total Cohort	Elective Surgery	Emergency Surgery
Effect-site concentration for induction: 4 mcg/mL						
mg	140 ± 42	152 ± 4.7	123 ± 29	33 ± 29	43 ± 30	18 ± 21
mg/kg	1.7 ± 0.3	1.8 ± 0.3	1.5 ± 0.2	0.4 ± 0.3	0.5 ± 0.3	0.2 ± 0.3
	*p*-value	0.015		*p*-value	0.271	
Effect-site concentration for induction: 6 mcg/mL						
mg	180 ± 47	190 ± 49	166 ± 40	19 ± 22	26 ± 24	9 ± 16
mg/kg	2.2 ± 0.3	2.3 ± 0.3	2.0 ± 0.2	0.2 ± 0.3	0.3 ± 0.3	0.1 ± 0.2
	*p*-value	0.079		*p*-value	0.287	
Effect-site concentration for induction: 8 mcg/mL						
mg	230 ± 58	239 ± 62	218 ± 54	13 ± 17	18 ± 19	7 ± 12
mg/kg	2.8 ± 0.2	2.8 ± 0.2	2.7 ± 0.2	0.2 ± 0.2	0.2 ± 0.2	0.1 ± 0.1
	*p*-value	0.223		*p*-value	0.517	

* Data are presented as the mean ± standard deviation.

**Table 2:** Propofol dosage administered (mg) one minute after peak of plasma concentration (t_0_) and x (t_x_) minutes after t_0_ (based on effect-site concentration at induction and reduction at t_0_ to maintenance effect-site concentration of 2.5 mcg/mL).

Propofol Dose *:	t_0_	t_1_	t_2_	t_3_	t_4_	t_5_	t_6_	t_7_	t_8_	t_9_	t_10_
Effect-site concentration for induction: 4 mcg/mL											
mg	107 ± 27	107 ± 27	107 ± 27	107 ± 28	113 ± 26	125 ± 28	136 ± 29	145 ± 30	155 ± 32	165 ± 33	175 ± 34
Effect-site concentration for induction: 6 mcg/mL											
mg	161 ± 41	161 ± 41	162 ± 42	162 ± 42	162 ± 42	162 ± 42	172 ± 38	181 ± 38	191 ± 39	200 ± 41	209 ± 42
Effect-site concentration for induction: 8 mcg/mL											
mg	217 ± 56	217 ± 56	218 ± 56	218 ± 56	218 ± 56	218 ± 56	218 ± 56	225 ± 53	234 ± 51	243 ± 52	251 ± 53

* Data are presented as the mean ± standard deviation.

## Data Availability

Data are available from the corresponding authors upon reasonable request.

## Abbreviations

The following abbreviations are used in this manuscript:

CD	Cesarean delivery
C_et_	Effect-site concentration
C_p_	Plasma concentration
PK/PD	Pharmacokinetic/pharmakodynamic
TCI	Target-controlled infusion

## References

[1] Delgado C., Ring L., Mushambi M.C. (2020). General Anaesthesia in Obstetrics. BJA Educ..

[2] Gwanzura C., Gavi S., Mangiza M., Moyo F.V., Lohman M.C., Nhemachena T., Chipato T. (2023). Effect of Anesthesia Administration Method on Apgar Scores of Infants Born to Women Undergoing Elective Cesarean Section. BMC Anesthesiol..

[3] Lee A., Estevez M.G., Le Gouez A., Mercier F.J. (2024). Cesarean Delivery: Clinical Updates. Best Pract. Res. Clin. Anaesthesiol..

[4] Afolabi B.B., Lesi F.E. (2012). Regional Versus General Anaesthesia for Caesarean Section. Cochrane Database Syst. Rev..

[5] Choi S.U. (2022). General Anesthesia for Cesarean Section: Are We Doing It Well?. Anesth. Pain Med..

[6] Desai N., Carvalho B. (2017). General Anaesthesia for Caesarean Section: Is the End in Sight for Thiopental?. Br. J. Hosp. Med..

[7] Desai N., Wicker J., Sajayan A., Mendonca C. (2018). A Survey of Practice of Rapid Sequence Induction for Caesarean Section in England. Int. J. Obstet. Anesth..

[8] Devroe S., Van de Velde M., Rex S. (2015). General Anesthesia for Caesarean Section. Curr. Opin. Anaesthesiol..

[9] Karim H.M.R. (2019). Thiopental Versus Propofol on the Outcome of the Newborn After Caesarean Section: Can Urgency Grade Affect the Impact?. Anaesth. Crit. Care Pain Med..

[10] Hedawy S., Labeeb E., Aldalahmeh S., Elswaf A., AlManaseer M., Shehab M.A., Menshawy A. (2025). Hypnotics as Induction Agents for General Anesthesia in Cesarean Section Patients: Updated Systematic Review and Meta-Analysis of Randomized Controlled Trials. J. Anesth..

[11] Jauniaux E., Gulbis B., Shannon C., Maes V., Bromley L., Rodeck C. (1998). Placental Propofol Transfer and Fetal Sedation During Maternal General Anaesthesia in Early Pregnancy. Lancet.

[12] Odor P.M., Bampoe S., Moonesinghe S.R., Andrade J., Pandit J.J., Lucas D.N. (2021). General Anaesthetic and Airway Management Practice for Obstetric Surgery in England: A Prospective, Multicentre Observational Study. Anaesthesia.

[13] Kimizuka M., Tokinaga Y., Azumaguchi R., Hamada K., Kazuma S., Yamakage M. (2021). Effects of Anesthetic Agents on Contractions of the Pregnant Rat Myometrium in Vivo and in Vitro. J. Anesth..

[14] Metodiev Y., Lucas D.N. (2022). The Role of Total Intravenous Anaesthesia for Caesarean Delivery. Int. J. Obstet. Anesth..

[15] Bidkar P.U., Dey A., Chatterjee P., Ramadurai R., Joy J.J. (2024). Target-Controlled Infusion—Past, Present, and Future. J. Anaesthesiol. Clin. Pharmacol..

[16] Schnider T.W., Minto C.F., Struys M.M., Absalom A.R. (2016). The Safety of Target-Controlled Infusions. Anesth. Analg..

[17] Nimmo A.F., Absalom A.R., Bagshaw O., Biswas A., Cook T.M., Costello A., Grimes S., Mulvey D., Shinde S., Whitehouse T. (2019). Guidelines for the Safe Practice of Total Intravenous Anaesthesia (Tiva): Joint Guidelines from the Association of Anaesthetists and the Society for Intravenous Anaesthesia. Anaesthesia.

[18] Schnider T., Minto C. (2008). Pharmacokinetic Models of Propofol for Tci. Anaesthesia.

[19] Watson S.E., Richardson A.L., Lucas D.N. (2022). Neuraxial and General Anaesthesia for Caesarean Section. Best Pract. Res. Clin. Anaesthesiol..

[20] Ren L.Q., Sun X.X., Guan Y. (2018). Effects of Sevoflurane or Propofol Combined with Remifentanil Anesthesia on Clinical Efficacy and Stress Response in Pregnant Women with Pregnancy-Induced Hypertension. Eur. Rev. Med. Pharmacol. Sci..

[21] Sumikura H., Niwa H., Sato M., Nakamoto T., Asai T., Hagihira S. (2016). Rethinking General Anesthesia for Cesarean Section. J. Anesth..

[22] Struys M.M., Sahinovic M., Lichtenbelt B.J., Vereecke H.E., Absalom A.R. (2011). Optimizing Intravenous Drug Administration by Applying Pharmacokinetic/Pharmacodynamic Concepts. Br. J. Anaesth..

[23] Errando C.L., Sigl J.C., Robles M., Calabuig E., García J., Arocas F., Higueras R., Del Rosario E., López D., Peiró C.M. (2008). Awareness with Recall During General Anaesthesia: A Prospective Observational Evaluation of 4001 Patients. Br. J. Anaesth..

